# Re-Vaccination in Prussia

**Published:** 1851-01

**Authors:** 


### *Re-Vaccination in Prussia.*

Re-vaccination is systematically practiced in Prussia. No child is admitted into a school without proof of vaccination, and every recruit is vaccinated on admission into the army. In the year 1848, twenty-eight thousand eight hundred and fifty-nine soldies were vaccinated; of these, the vaccine disease was regular in sixteen thousand eight hundred and eighty-two; in four thousand four hundred and four individuals it was irregularly developed; and in seven thousand five hundred and seventy-three it did not take any effect.—*London Medical Gazette.*

